# Modifications of the Aerobic Respiratory Chain of Paracoccus Denitrificans in Response to Superoxide Oxidative Stress

**DOI:** 10.3390/microorganisms7120640

**Published:** 2019-12-03

**Authors:** Vojtěch Sedláček, Igor Kučera

**Affiliations:** Department of Biochemistry, Faculty of Science, Masaryk University, Kotlářská 2, 61137 Brno, Czech Republic

**Keywords:** NADH dehydrogenase, succinate dehydrogenase, terminal oxidase, iron–sulfur cluster, FnrP transcription factor, superoxide

## Abstract

*Paracoccus denitrificans* is a strictly respiring bacterium with a core respiratory chain similar to that of mammalian mitochondria. As such, it continuously produces and has to cope with superoxide and other reactive oxygen species. In this work, the effects of artificially imposed superoxide stress on electron transport were examined. Exposure of aerobically growing cells to paraquat resulted in decreased activities of NADH dehydrogenase, succinate dehydrogenase, and *N*,*N*,*N*’,*N*’-tetramethyl-p-phenylenediamine (TMPD) oxidase. Concomitantly, the total NAD(H) pool size in cells was approximately halved, but the NADH/NAD^+^ ratio increased twofold, thus partly compensating for inactivation losses of the dehydrogenase. The inactivation of respiratory dehydrogenases, but not of TMPD oxidase, also took place upon treatment of the membrane fraction with xanthine/xanthine oxidase. The decrease in dehydrogenase activities could be fully rescued by anaerobic incubation of membranes in a mixture containing 2-mercaptoethanol, sulfide and ferrous iron, which suggests iron–sulfur clusters as targets for superoxide. By using cyanide titration, a stress-sensitive contribution to the total TMPD oxidase activity was identified and attributed to the *cbb*_3_-type terminal oxidase. This response (measured by both enzymatic activity and mRNA level) was abolished in a mutant defective for the FnrP transcription factor. Therefore, our results provide evidence of oxidative stress perception by FnrP.

## 1. Introduction

Respiratory chains of mitochondria, bacteria and archaea are a series of membrane-associated enzymes, called respiratory complexes, that perform electron transfer from reduced substrates such as NADH or succinate to dioxygen, which is converted to two molecules of water in a four-electron process catalyzed by the terminal oxidase. Energy derived from these redox reactions is conserved into a protonmotive force across the membrane and then utilized by ATP synthase to generate ATP. However, dioxygen can also undergo stepwise reduction, electron by electron, at other sites of the chain. Such uncontrolled side reactions lead to the formation of harmful superoxide and other reactive oxygen species (ROS) that compromise cell viability by interacting with DNA, RNA, lipids and proteins.

In addition to being important sources of ROS in cells [1,2,3], the respiratory complexes are also vulnerable targets to oxidative damage. Working with bovine heart submitochondrial particles, Zhang et al. [4] demonstrated that hydroxyl radical rapidly inactivated both NADH dehydrogenase and succinate dehydrogenase, whereas superoxide acted preferentially on the former enzyme. In the isolated rat liver mitochondria, the superoxide generator paraquat induced multiple effects on oxidative phosphorylation, including depression of respiratory activity through partial inhibition at ubiquinol-cytochrome *c* reductase and cytochrome *c* oxidase [5]. The breakdown of respiratory electron flow due to ROS attack was identified as the key event in the inactivation of *Escherichia coli* by UVA irradiation [6] or by tellurite treatment [7].

*Paracoccus denitrificans*, an α-proteobacterium considered to be related to the protomitochondrion [8], possesses an aerobic respiratory chain closely resembling the mitochondrial one. Its respiratory complexes I, III, and IV are, however, constituted by a smaller number of subunits—14, 3 and 4 [9,10,11], respectively—compared with 46, 11, and 13 subunits of the corresponding bovine complexes [12]. For this reason, the bacterium has become a significant focus in bioenergetic research. A significant difference in comparison with mitochondria is the utilization of multiple terminal oxidases. The traditional *aa*_3_-type cytochrome *c* oxidase is encoded by the *ctaCBGE* operon (*pden_4321* to *4317*), containing genes for subunits II and III, by the single genes *ctaDI* (*pden_3028*) and *ctaDII* (*pden_1938*) for isoforms of subunit I and by the *pden_0432* gene for subunit IV. The activity of *ctaDII* and *ctaC* promoters is most pronounced under high aeration [13]. The *cbb*_3_-type cytochrome c oxidase is encoded in the *ccoNOQP* operon (*pden_1848* to *1845*) which is expressed at a low level under aerobic conditions and induced substantially under low oxygen tension [13,14]. The *ba*_3_-type quinol oxidase is the product of the *qoxABCD* operon (*pden_5108* to *5105*). Its expression is similar at both low and high oxygen levels and increases in the presence of nitrate [13,15]. Due to its rather high Michaelis constant for ubiquinol, the enzyme becomes active only at a higher degree of reduction of the ubiquinone pool [16]. The *P. denitrificans* genome also encodes a putative second quinol oxidase, possibly of the cytochrome *bd* type (*pden_4010* and *4011*), but experimental evidence on its in vivo functioning is still lacking.

The response of *P. denitrificans* to variation in cellular oxygen status is mediated by the FnrP transcription factor which senses oxygen by a cubane iron–sulfur cluster [17]. Oxidative disassembly of the cluster in the presence of oxygen causes FnrP to lose affinity for DNA. The relative slowness of the disassembly process [18] allows FnrP to be transcriptionally active not only under anaerobic but also under microaerobic conditions. FnrP binding sites are present upstream of the *ccoN* and *qoxA* genes and the observed phenotypic alterations in *fnrP* mutant [13,19] suggest that FnrP serves as a positive regulator of the *cco* operon and a negative regulator of the *qox* operon.

Being a strict respirer, *P. denitrificans* has to continuously cope with the generated ROS. This, along with its mitochondrion-like features [20], make it potentially useful as a model organism in oxidative stress research. Interestingly, despite extensive knowledge on the structure and function of the respiratory chain components, the effects of oxidative stress on aerobic respiration in this bacterium have not been investigated so far. In the present paper, we specifically address this problem by using paraquat (*N*,*N*′-dimethyl-4,4′-bipyridinium dichloride) and xanthine oxidase with xanthine as external sources of superoxide for in vivo and in vitro experiments, respectively.

## 2. Materials and Methods 

### 2.1. Bacterial Strains and Growth Conditions

*Paracoccus denitrificans* strains have the following characteristics: Pd1222 (parental strain, Rif^r^ [21]), Pd2921 (*fnr*P::Km^r^, [19]), Pd9233 (Δ*cco*NO, *qox*B::Km^r^ [13]), Pd9311 (Δ*cta*DI, Δ*cta*DII, *qox*B::Km^r^ [14]). The basal growth medium was composed of 17 mM Na_2_HPO_4_, 33 mM KH_2_PO_4_, 50 mM NH_4_Cl, 1 mM MgSO_4_, 30 µM ferric citrate and 50 mM succinate and was adjusted to pH 7.3 before subjecting to sterilization. Cell growth was performed aerobically in 3 L conical flasks with 500 mL bacterial culture at 30 °C and 250 rpm for 16 h. A volume of 15 mL pre-grown culture was taken as the inoculum. Stationary-phase cells were harvested by centrifugation at 4600× *g* for 30 min at 4 °C.

### 2.2. Membrane Preparation and Protein Determination

Cytoplasmic membrane vesicles were prepared by lysozyme treatment of bacterial cells and osmotic lysis [22] and were stored in a frozen state at −25 °C. Protein concentration was determined using the Pierce BCA Protein Assay Kit (Thermo Fisher Scientific, Waltham MA, USA, [23]). Prior to incubation with working reagent, 0.01 mL of concentrated membrane samples were heated at 100 °C for 30 min in the presence of 1 M NaOH in a total volume of 0.5 mL.

### 2.3. Determination of Succinate

The amount of succinate remaining in the culture media was quantified as dissolved organic carbon (TC/NPOC) using a total carbon analyzer (Multi N/C 2100 S, Analytik Jena AG, Jena, Germany).

### 2.4. Enzyme Activities

Spectrophotometric assays were carried out at 30 °C on an UltroSpec 2000 spectrophotometer (GE Healthcare Pharmacia Biotech, Uppsala, Sweden) in 1 cm cuvettes containing 2 mL of 0.1 M sodium phosphate buffer of pH 7.3. NADH dehydrogenase was measured at 340 nm (ε = 6.22 mM^−1^ cm^−1^) with 0.1 mM NADH and 0.25 mM K_3_[Fe(CN)_6_]. Succinate dehydrogenase was measured at 578 nm (ε = 20.5 mM^−1^ cm^−1^) using 20 mM succinate as electron donor, 0.05 mM 2,6-dichlorophenolindophenol (DCIP) as electron acceptor and 0.05 mM phenazine methosulfate (PMS) as mediator. 2.5 mM NaCN was added to both dehydrogenase assays to inhibit terminal oxidases. *N*,*N*,*N*′,*N*′-tetramethyl-p-phenylenediamine (TMPD) oxidase was measured by monitoring the formation of the Wurster’s blue (WB) cation-radical at 612 nm (ε = 12.7 mM^−1^ cm^−1^) nm in the presence of 0.3 mM TMPD. Oxygen uptake due to NADH and succinate oxidation was followed amperometrically in a closed magnetically stirred vessel of 3 mL total volume, tempered to 30 °C and fitted with a Clark-type oxygen sensor. The reaction mixture contained 0.2 mM NADH or 5 mM succinate in 0.1 M sodium phosphate (pH 7.3). Data were obtained with an EmStat potentiostat operated by the PalmSensLite software (Palm Instruments BV, Houten, The Netherlands). All activity measurements were conducted in triplicate and error bars represent the standard deviation (SD).

### 2.5. Superoxide Production Assay

Generation of superoxide was assessed semi-quantitatively by measuring the fluorescence of cells treated by hydroethidine, a fluorogenic probe [24]. The probe was added at a final concentration of 200 µM to 200 µL 9-hour-old microplate cultures, grown at 30 °C and 240 rpm on an ELx808 microplate reader (BioTek, Winooski, VT, USA). After an additional 15 min of growth, 150 µL of the culture was withdrawn, diluted in 0.1 M sodium phosphate buffer (pH 7.3) to a final volume of 3 mL and the fluorescence of the suspension was measured in a 1 cm cuvette in an LS-50 fluorimeter (Perkin-Elmer. Ltd., Beaconsfield, England) using an excitation wavelength of 515 nm, an emission wavelength of 578 nm, and slit widths of 5 nm for excitation and 20 nm for emission. The fluorescence signal was normalized to a cell concentration of 10^7^ per mL to account for the differences in growth behavior.

### 2.6. In Vitro Superoxide Exposure

For examination of the direct effect of superoxide on membrane-associated enzyme activities, the membranes (26.6 mg protein) were incubated with xanthine (0.1 mM) and xanthine oxidase (10.4 mU) at 25 °C in a 0.6 mL volume of 0.1 M sodium phosphate buffer (pH 7.3). The mixture was stirred in a beaker on a magnetic stirrer and 10 µL samples were periodically withdrawn for activity measurement. Control samples without membranes were also analyzed in order to exclude possible background reactions. The involvement of superoxide was confirmed by addition of superoxide dismutase (130 U).

### 2.7. Iron Sulfur Clusters Restoration

The procedure used is a slight modification of previously described protocols [7,25]. A solution was prepared containing 0.1 M NaCl, 0.1 M 2-mercaptoethanol, 0.2 mM Na_2_S and 0.1 M sodium phosphate buffer (pH 7.3) in which membranes were suspended at the final concentration of 0.5 mg protein per mL. After bubbling with argon for 10 min to remove the dissolved oxygen, (NH_4_)_2_Fe(SO_4_)_2_ was added up to 0.25 mM and the mixture was incubated on ice for 60 min. Finally, the membranes were separated by ultracentrifugation (40 min, 70,400× *g*, 4 °C) and resuspended in argon-saturated pure buffer. In a control experiment, (NH_4_)_2_Fe(SO_4_)_2_ was omitted from the restoration buffer.

### 2.8. Intracellular Concentrations of NAD^+^ and NADH

NAD^+^ and NADH concentrations in control and stressed cells were determined by adapting an enzymatic cycling assay described previously [26,27]. A starting culture with an OD_600_ of 0.2 from the Pd1222 strain was divided into two equal portions, and 50 µM of paraquat was added to one of them and cells were allowed to grow for 7 h. After that, aliquots of 20 mL were withdrawn, and cell pellets obtained upon centrifugation at 10,000× *g* for 5 min at 0 °C were frozen and kept at −80 °C until further analysis. Each pellet was thawed and suspended in 1 mL of either 0.2 M HCl (for NAD^+^ determination) or 0.2 M NaOH (for NADH determination). 0.9 mL samples of the suspensions were heated to 55 °C for 10 min, neutralized by 0.9 mL of 0.2 M NaOH or 0.2 M HCl, and cleared by centrifugation at 12,000× *g* for 5 min at 0 °C. The NAD(H) assay was conducted by mixing 0.5 mL sample and 0.1 mL each of 1 M Bicine-NaOH (pH 8.0), 4.2 mM thiazolyl blue tetrazolium bromide (MTT), 16.6 mM phenazine methosulfate (PMS), 17.2 M ethanol, and 40 mM EDTA. After 3 min preincubation at 30 °C, 20 µL of 550 U/mL of yeast alcohol dehydrogenase (Sigma-Aldrich, St Louis MO, USA, A3263) was added, and the increase in absorbance at 570 nm due to formazan formation was monitored. The amount of NAD(H) present was calculated using a calibration curve based on the slopes of absorbance–time recordings obtained for an NAD^+^ standard in the range of 0 to 1000 pmol. For conversion to intracellular concentrations, an intracellular volume of 2.7 µL per mg dry weight was assumed [28]. The results presented are the mean ± SD of three technical replicates.

### 2.9. qRT-PCR

Next, 100 mL conical flasks containing 15 mL of the succinate mineral medium were inoculated at an initial OD_600_ of 0.1 units with overnight pre-cultures of either the parental or the *fnrP*^-^ strain and incubated at 30 °C while shaking at 250 rpm in an orbital shaker. When the culture reached an OD_600_ of 0.2, paraquat in water (or water alone) was added to a final concentration of 50 µM and growth was allowed to continue until an OD_600_ of 0.6 had been attained. Cells from 1 mL of culture were then harvested by centrifugation at 11,000× *g* for 1 min and stored at −80 °C or immediately processed further. Total RNA was extracted with TRIzol reagent (Top-Bio, Prague, Czech Republic) and cleared of DNA contamination with a TURBO Ambion DNAfree kit (ThermoFisher Scientific, Waltham MA, USA). 1.25–1.5 µg of purified RNA from each sample was reversely transcribed to cDNA with random hexamer primers (ThermoFisher Scientific, Waltham MA, USA) and M-MLV reverse transcriptase (Top-Bio, Prague, Czech Republic) according to the manufacturer’s instructions. Relative levels of mRNA transcripts coding for the catalytic subunits of *aa*_3_-type cytochrome *c* oxidase (*ctaDII*, *pden_1938*), *cbb*_3_-type cytochrome *c* oxidase (*ccoN*, *pden_1848*) and the *ba*_3_-type quinol oxidase (*qoxB*, *pden_5107*) were estimated by quantitative PCR on a Light Cycler 480 (Roche, Basel, Switzerland) with the following gene-specific primers FP_qPCR_1938 (5′-GCCTGATCTCGGTATGCTTC-3′), RP_qPCR_1938 (5′-CGTGGTAGGTGATCATGACG-3′), FP_qPCR_1848 (5′-ACCTGTCCTTCATCGTCACC-3′), RP_qPCR_1848 (5′-ATACATCATGCCCAGGAAGC-3′), FP_qPCR_5107 (5′-GATGGTCTATGCCACGGTCT-3′), RP_qPCR_5107 (5′-TCACGAAGGTCAGCATGAAG-3′). The housekeeping genes examined were glyceraldehyde 3-phosphate dehydrogenase (*pden_1060*, forward primer – 5’-TTTCCTCGGACTTCAACCAC-3´, reverse primer – 5´-CTCGTTGTCATACCAGGTCAG-3´), sigma factor 54 (*pden_2604*, forward primer – 5´-GGCTCAAGGATCACTACAAGG, reverse primer – 5´-TCCTGCGTTTCCCATTCATC-3´) and beta subunit of DNA polymerase III (*pden_0970*, forward primer – 5´-GTGCCTATCCCGATTACACG-3´, reverse primer – 5´-TCTAAATCGAACTTCAGGGCG-3´). One reaction mixture contained 2 µL cDNA equivalent to 5–6 ng of total RNA, 4 µL RNase-free water (Top-Bio, Prague, Czech Republic), 2 µL (10 pmol) forward primer, 2 µL (10 pmol) reverse primer, and 10 µL 2x SYBR Master Mix (Top-Bio, Prague, Czech Republic). After an initial polymerase activation step at 95 °C for 15 min, PCR amplification was performed as follows: 40 cycles of DNA denaturation and annealing at 95 and 60 °C for 20 s and extension at 72 °C for 30 s, with three replicates for each sample analyzed. In each experiment, the C_t_ (cycle threshold) values for the gene of interest were normalized to the C_t_ of three housekeeping genes and then averaged. The relative mRNA expression was calculated as fold change 2^−ΔΔCt^ [29].

## 3. Results

Paraquat at concentrations of the order of ten micromolar deteriorates the aerobic growth of *P. denitrificans* both in terms of growth rate and maximum cell density [30], which indicates that these concentrations are effective in inducing oxidative stress. When 50 µM paraquat was added at the beginning of the cultivation, the maximum biomass concentration attained in the stationary phase was 0.9 g/L, which is lower compared to the value of 1.3 g/L found in the absence of paraquat. At the end of cultivation, the growth substrate succinate was not totally depleted but there was a remaining amount of at least 20 mM in either culture. This evidenced that the cultures were not carbon limited.

The paraquat dication is known to undergo a one-electron reduction, forming a monocation radical that reacts with oxygen to produce superoxide and regenerate the original dication species [31]. Superoxide formation was detected in a separate growth experiment on 96-well plates using hydroethidine as a fluorogenic probe. When 50 µM paraquat was included in the growth medium, the normalized fluorescence signal obtained from the probe at 15 min after its addition was approximately threefold as compared to the cultures lacking paraquat (680 ± 50 vs. 240 ± 60; mean ± SD, 3 replicates). The paraquat effect was completely abolished when 130 U/mL superoxide dismutase was also present (130 ± 20; mean ± SD, 3 replicates), confirming that paraquat cycling with superoxide production indeed took place in our experimental conditions.

**Figure 1:**
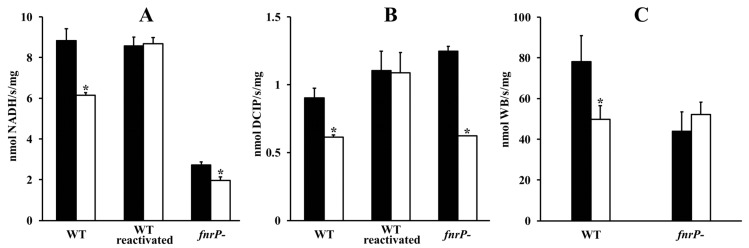
Specific activities of membrane-bound respiratory enzymes. (**A**) NADH dehydrogenase (NADH: ferricyanide oxidoreductase); (**B**) succinate dehydrogenase (succinate: DCIP oxidoreductase); (**C**) TMPD oxidase. Black and white bars indicate that the cells from which the membranes were derived were grown in the absence and presence of 50 µM paraquat, respectively. The activities in the wild-type (WT) strain membranes were determined before and after chemical reconstitution of iron–sulfur clusters. Bars represent the mean ± SD for three measurements. Asterisk denotes a statistically significant difference (* *p* < 0.05) with regard to membranes from cells grown in the absence of added paraquat.

**Figure 2:**
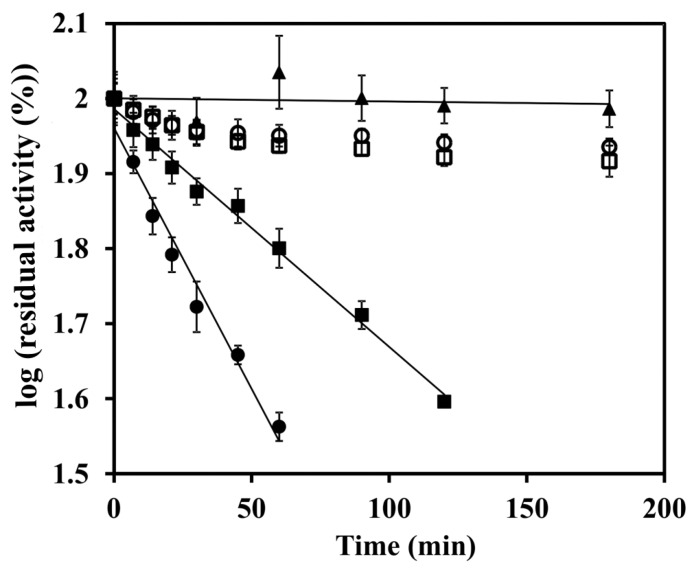
Log(activity)–time plot for determining the rate constants for the inactivation by superoxide of NADH dehydrogenase (circles), succinate dehydrogenase (squares), and TMPD oxidase (triangles). Filled and open symbols represent the absence and the presence of 650 U/mL superoxide dismutase. Each data point is a mean of triplicate determinations ± SD.

Membrane vesicles prepared from the stationary-phase cells exhibited enzyme activities summarized in Figure 1. It can be seen that paraquat-induced oxidative stress in growing cells resulted in a decreased efficiency of the electron transport chain, as demonstrated by the decrease in the specific activities of NADH dehydrogenase, succinate dehydrogenase and cytochrome *c*-dependent TMPD oxidase. Somewhat different results were obtained when the inactivation of respiratory enzymes was evaluated in a cell free system consisting of purified membranes and a mixture of xanthine oxidase/xanthine as an alternative source of reactive oxygen species. In this case, both dehydrogenase activities decreased gradually after the onset of the stress while TMPD oxidase remained unaffected. Analysis of the initial phases of inactivation curves with first order kinetics (Figure 2) yielded rate constants of (2.7 ± 0.2) × 10^−4^ s^−1^ and (1.22 ± 0.04) × 10^−4^ s^−1^ for NADH dehydrogenase and succinate dehydrogenase, respectively, indicating an approximately twofold greater sensitivity of the former enzyme. Inclusion of superoxide dismutase (650 U/mL) afforded greater than 80 percent protection to the dehydrogenases from inactivation. This proved superoxide to be the key inactivation agent. NADH and succinate oxidases (measured as oxygen consumption) displayed the same kinetics of inactivation as the respective dehydrogenases, which implies that the same inhibition mechanism was operating.

The previous findings raised the question of the reversibility of the inactivation process. We attempted to restore dehydrogenase activities by adopting a procedure used earlier for the reconstruction of the iron–sulfur clusters in soluble [25] and membrane [7] enzymes. After 60 min anaerobic incubation with mercaptoethanol, Fe^2+^ and S^2-^, the enzymatic activities of membranes prepared from cells grown with paraquat were found to be increased by approximately 1.4–1.8 fold to levels comparable with those of membranes from paraquat-untreated cells (Figure 1). The omission of ferrous iron from the mixture precluded activity recovery. The virtually complete reactivation under the above specific conditions suggests that it was the iron–sulfur clusters of the dehydrogenases that were damaged during the superoxide stress. 

**Figure 3:**
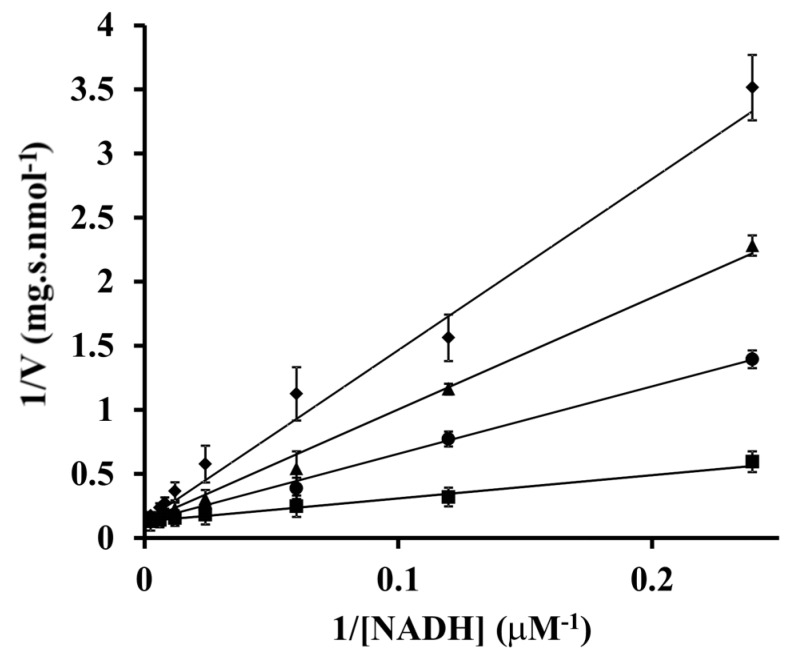
Initial velocity analysis of NADH dehydrogenase inhibition by NAD^+^. Experimental data are shown as symbols in the double-reciprocal plots and lines indicate the global fit of all data to the equation for competitive inhibition. The concentrations of NAD^+^ used were as follows: 0 µM, squares; 300 μM, circles; 600 μM, triangles; 1000 μM, rhombs. Each data point is a mean of triplicate determinations ± SD.

Having identified the respiratory NADH dehydrogenase as a target of oxidative insult, we next analyzed the actual changes in NADH/NAD^+^ levels in cells subjected to 50 µM paraquat. We saw a marked drop in NAD^+^ concentration from 750 ± 30 µM to 330 ± 20 µM. The concentration of NADH also decreased, but only minimally (from 37 ± 5 µM to 32 ± 1 µM). As a result, the total NAD(H) pool size decreased to 46 % and the degree of NAD reduction increased from 4.7% to 8.8%. To find out how these concentration changes could affect the rate of NADH oxidation by the respiratory chain, the kinetic parameters for NADH oxidase were evaluated in a cell-free preparation of plasma membrane vesicles (Figure 3). The Michaelis constant (*K*_M_) for NADH and maximum velocity (*V*_max_) were found to be 14 ± 2 µM and 7.8 ± 0.2 nmol s^−1^ mg protein^−1^ respectively. The reaction product, NAD^+^, acted as a competitive inhibitor with an inhibition constant (*K*_i_) of 160 ± 20 µM. In contrast to NAD^+^, NADP^+^ in a millimolar concentration did not inhibit the reaction at all. From the above values it can be estimated that the fractional saturation of the enzyme by NADH, expressed as 100×([NADH]/*K*_M_)/(1+([NADH]/*K*_M_)+ ([NAD^+^]/*K*_i_)) was higher in paraquat treated cells compared to untreated controls (42% vs. 31%).

**Figure 4:**
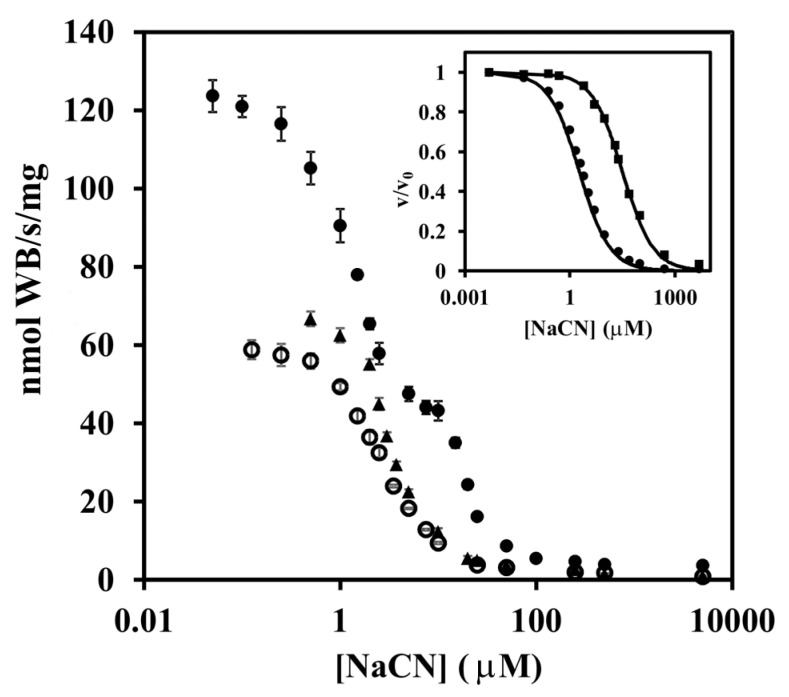
Titration of TMPD oxidase activity with NaCN. Close circles, membranes from wild-type cells grown without paraquat; open circles, membranes from wild-type cells grown with 50 µM paraquat; close triangles, membranes from *fnrP* mutant cells grown without paraquat. The inset shows cyanide titrations of membranes from strains Pd9233 (circles) and Pd9311 (squares) that contain cytochrome *aa*_3_ and *cbb*_3_, respectively, as the sole terminal oxidase. Each data point is a mean of triplicate determinations ± SD. Solid lines are best fit to the equation *v*/*v*_0_ = 1/(1 + ([I]/IC_50_)).

The following experiments investigated possible reasons why only in vivo and not in vitro oxidative stress was effective in changing the activity level of TMPD oxidase. TMPD funnels electrons through cytochrome *c* to two terminal oxidases (*aa*_3_- and c*bb*_3_-type). To dissect the contributions coming from individual enzymes, a titration with cyanide was conducted (Figure 4). The titration curve for membranes from cells grown in the absence of paraquat was biphasic; two-thirds of the activity was inhibited at cyanide concentrations lower than 7.5 μM, while the rest was inhibited at 10–100 μM. In contrast, cells grown with paraquat gave essentially monophasic titration curve indicating the sole presence of a terminal oxidase with high sensitivity to cyanide. From the titration data obtained with membranes of the single-oxidase strains Pd9233 and Pd9311 (the inset in Figure 4), we determined the half-maximal inhibitory concentration to be 2.32 ± 0.03 μM for the *aa*_3_-type cytochrome *c* oxidase and 33 ± 1 μM for the *cbb*_3_-type cytochrome *c* oxidase. A combination of these results allowed us to conclude that oxidative stress selectively suppressed the synthesis of the *cbb*_3_ enzyme. This was further supported by the finding that the specific TMPD oxidase activity in the *cbb*_3_ single-route strain Pd9311 grown with paraquat was only approximately 2.7% of that of the control grown without the stressor.

**Figure 5:**
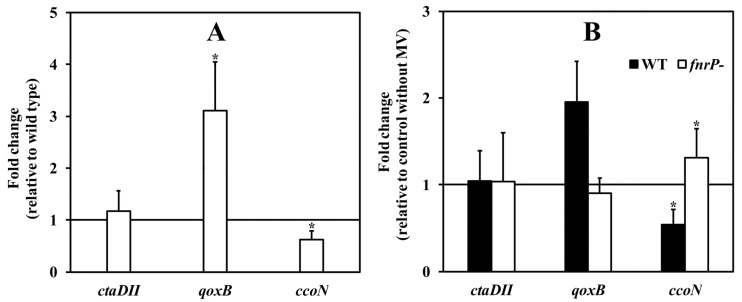
Transcription level analysis of genes for catalytic subunits of terminal oxidases. (**A**), the effect of *fnr* gene inactivation; (**B**), the effect of paraquat on wild-type strain (black bars) and *fnrP* mutant strain (white bars). Bars represent the mean ± SD for three measurements. Asterisk denotes a statistically significant difference (* *p* < 0.05) from 1.

Next, the possible involvement of the FnrP protein in the response of the terminal region of the respiratory chain to oxidative stress was examined. When we performed the above mentioned measurements with the FnrP-deficient strain Pd2921, grown either in absence or presence of paraquat, we found that in both cases the level of TMPD oxidase activity in membranes (Figure 1) and the shape of the titration curve for cyanide inhibition (Figure 4) were essentially the same as those for the wild-type cells grown with paraquat. In another approach, we employed the relative quantification of the cellular mRNA expression of the genes *pden_1938*, *pden_5107*, and *pden_1848*, coding for catalytic subunits CtaDII, QoxB, and CcoN of the *aa*_3_, *ba*_3_, and *cbb*_3_ oxidases (Figure 5). As expected, FnrP mutation increased the level of *pden_5107* mRNA, decreased the level of *pden_1848* mRNA, and did not affect the expression of *pden_1938* (Figure 5A). The same effect was produced by paraquat in wild-type but not mutant cells (Figure 5B), which clearly demonstrated the participation of FnrP in signal transfer.

## 4. Discussion

Based on the results above, iron–sulfur clusters damage emerges as a common mechanism underlying superoxide-induced alterations in electron transport activities of *P. denitrificans* respiratory enzymes. This is in line with the general notion that superoxide exerts its toxic effect primarily by oxidizing [4Fe–4S]^2+^ clusters, causing the release of ferrous ion and the production of catalytically inactive forms [3Fe–4S]^+^ [32]. Previous studies on *E. coli* have established that whereas the [4Fe–4S] clusters of soluble hydro-lyases such as aconitase are rapidly destroyed by superoxide (reviewed in [33,34]), the clusters of the membrane-bound respiratory NADH dehydrogenase I and succinate dehydrogenase withstand a pulse of oxidant without loss of activity [35]. The dehydrogenases of *P. denitrificans* appear to be more susceptible to superoxide inactivation and resemble in this respect the mitochondrial enzymes [4]. The successful in vitro reactivation suggests that the respiratory complexes remain in a fairly preserved state in the membrane after the superoxide radical attack and are possibly continuously repaired in the cell. 

It would seem that the partial inactivation of NADH dehydrogenase should bring about a rise in NADH concentration. Because a slight decrease was seen instead, it is probable that some endogenous processes generating NADH are also affected, which then limits NADH supply. Succinate is catabolized by *P. denitrificans* via the tricarboxylic acid cycle in combination with malic enzyme and pyruvate dehydrogenase producing acetyl-CoA necessary for the cycle operation [36]. In addition to succinate dehydrogenase identified here, other proteins involved in these pathways could be damaged by paraquat-generated stress—the most likely candidate being the aconitase enzyme.

The most striking effect observed here was an approximate halving of the NAD^+^ content in the cells. Similar changes in nicotinamide cofactor concentrations upon stress exposure have been reported previously for other bacteria and ascribed mainly to the phosphorylation of NAD^+^ to NADP^+^ by an NAD kinase (reviewed in [37]). A link between oxidative stress and a decline in NAD^+^ levels has also been shown for human tissues and correlated with an increased activity of poly(ADP-ribose) polymerase [38]. Our data suggest that the increase in the NADH/NAD^+^ ratio leads to a greater saturation of the NADH dehydrogenase, which partly compensates for its loss due to inactivation. Moreover, the NADH/NAD^+^ ratio may serve as a regulatory signal for synthesis of some proteins. This would explain our earlier apparently contradictory finding that the *pden_5119* gene encoding a soluble NADH oxidase is expressed in response not only to external oxidative stress agents but also to respiratory chain inhibitors and terminal oxidase downregulation [39].

Our results also support superoxide as a physiologically relevant modulator of the oxygen sensor FnrP. Oxygen sensing by FNR proteins involves single-electron transfer between their [4Fe–4S]^2+^ cluster and an O_2_ molecule and subsequent dissociation of dimeric FNR into transcriptionally inactive, [2Fe–2S]^2+^ cluster-containing monomers. The superoxide formed by oxygen reduction is believed to be rapidly recycled back to oxygen by the combined actions of superoxide dismutase and catalase [40]. We propose that increased levels of superoxide promote dimer-to-monomer conversion by more effective oxidation of the [4Fe–4S]^2+^ cluster compared to the reaction with O_2_. The dimer/monomer ratio can be further shifted towards monomer by superoxide-mediated degradation of the [2Fe–2S]^2+^ cluster, yielding a cluster-less apo-form of FNR [41]. The above reported changes in enzyme activity and mRNA levels in paraquat treated wild-type and FnrP mutant cells corresponds with our earlier observation of a decreased content of the Pden_1845–1848 proteins [42] and, taken together, provide evidence for concluding that the inability to synthetize the *cbb*_3_-type terminal oxidase is due to FnrP inactivation by superoxide. Since the *cbb*_3_-type terminal oxidases are characterized by their high affinity for O_2_ [43], a physiological consequence of this enzyme deficiency may be poor growth under oxygen limitation. Judging from available proteomic data [44,45], the FnrP regulon includes tens of genes—the expression of each of which could in principle be affected by superoxide. The destruction of the [4Fe–4S]^2+^ cluster by superoxide could thus be an alternative to another mechanism of superoxide sensing which is mediated by another transcription factor (SoxR) and relies on reversible oxidation of a [2Fe–2S]^+^ cluster (reviewed in [46]).

In summary, these data identify respiratory NADH dehydrogenase as the main target for direct superoxide attack among the respiratory complexes of *P. denitrificans*. In vivo, the inactivation defect is partly compensated by decreased product inhibition due to a marked lowering of NAD^+^ concentration in superoxide-stressed cells. We also demonstrate that electron transfer in the branched terminal part of the respiratory chain is modulated by superoxide at a transcriptional level. The common denominator of all these superoxide effects appears to be oxidative damage to the iron–sulfur centers of the involved proteins.
